# Meningioma: Novel Diagnostic and Therapeutic Approaches

**DOI:** 10.3390/biomedicines13030659

**Published:** 2025-03-07

**Authors:** Carlen A. Yuen, Michelle Zheng, Max A. Saint-Germain, David O. Kamson

**Affiliations:** 1 Chao Family Comprehensive Cancer Center, University of California, Irvine, CA 92697, USA; 2 Division of Neuro-Oncology, Department of Neurology, University of California, Irvine, CA 92697, USA; 3 Charlie Dunlop School of Biological Sciences, University of California Irvine, Irvine, CA 92697, USA; 4 The Sidney Kimmel Comprehensive Cancer Center, Johns Hopkins University, Baltimore, MD 21231, USA; 5 Department of Neurology, School of Medicine, Johns Hopkins University, Baltimore, MD 21205, USA

**Keywords:** meningioma, 177-Lutetium, PRRT, DOTATATE PET, octreotide, *NF2*, *SMO*, *AKT*, vismodegib, abemaciclib, capivasertib

## Abstract

**Background/Objectives**: Meningiomas are the most common intracranial tumors. Surgery and radiation therapy are the cornerstones of treatment and no standard of care therapy exists for refractory meningiomas. This manuscript aims to provide a comprehensive review of novel diagnostic and therapeutic approaches against these tumors. **Methods**: A search for the existing literature on systemic therapies for meningiomas was performed on PubMed and a search for presently accruing clinical trials was performed on ClinicalTrials.gov. **Results**: Systemic treatments, including chemotherapy, somatostatin analogs, anti-hormone therapy, and anti-angiogenic therapy, have been extensively studied with marginal success. Targeted therapies are actively being studied for the treatment of meningiomas, including focal adhesion kinase (FAK), sonic hedgehog signaling pathway, phosphoinositide-3-kinase (*PI3K*), and cyclin-dependent kinases (*CDK*) inhibitors. These driver mutations are present only in a subset of meningiomas. In stark contrast, somatostatin receptor 2 (SSTR2) is ubiquitously expressed in meningiomas and was formerly targeted with somatostatin analogs with modest success. Theranostic SSTR2-targeting via [^68^Ga]DOTATATE for PET imaging and β-emitting [^177^Lu]DOTATATE for the treatment of meningiomas are currently under active investigation. **Conclusions**: A nuanced approach is needed for the treatment of refractory meningiomas. Targeted therapies show promise.

## 1. Introduction

Meningiomas are the most common adult tumor entity originating in the central nervous system (CNS). Meningiomas account for 41.7% of CNS tumors, occurring in 10.15 per 100,000 of the population, with an incidence on the rise [1]. Meningiomas of any grade occur more frequently in those of non-Hispanic Black ethnicity compared to non-Hispanic White ethnicity [1]. For non-malignant meningiomas, there is a higher incidence among those of non-Hispanic White ethnicity, while a higher incidence of malignant meningiomas is found in non-Hispanic Asian or Pacific Islanders [1]. Further, meningiomas predominantly occur in females and in those of advanced age >65 years [1,2,3]. Sex-specific molecular differences may exist, with increased aggressive biologic behavior in the meningiomas of female patients with chromosome X loss [4]. Spinal meningiomas account for 40.2% of all spine tumors among patients >20 years of age [1]. Furthermore, anatomic location has apparent molecular underpinnings. *NF2* mutant meningiomas typically occur along the convexity, while smoothened (*SMO*) mutated meningiomas are located within the olfactory groove [5]. *TRAF7* and *AKT1* alterations are associated with the anterior skull base [5]. The only established acquired risk factor for the development of meningiomas is ionizing radiation, as evidenced in atomic bomb survivors and those who received radiation to the scalp for tinea capitis or intracranial tumors [6,7]. While obesity has also been associated with meningioma development, the risk of meningioma with exogenous hormones remains controversial [7,8,9]. Genetic risk factors include predisposition syndromes associated with driver mutations, including *NF2* (NF2-related schwannomatosis), *PTCH1* (Gorlin Syndrome), *PTEN* (Cowden Syndrome), *SMARCE1*, and *BAP1* [10,11].

Meningioma patients present with a wide range of neurological symptoms, and meningiomas are provisionally diagnosed with brain imaging [12]. While computed tomography (CT) may identify calcification that negatively correlates with growth rate, magnetic resonance imaging (MRI) T2 hyperintensity positively correlates with growth rate in meningiomas [13]. In addition, meningiomas may demonstrate peritumoral edema, particularly in the angiomatous, microcystic, and secretory histologic subtypes [14]. Meningiomas are typically homogeneous, contrast-enhancing, and may reveal a characteristic dural tail [14]. CT or MR angiography is often obtained to evaluate the surrounding vasculature and the need for pre-operative embolization to minimize intraoperative blood loss [15].

Preceding the 2021 WHO Classification of CNS Tumors, meningioma grade was solely reliant on histopathological criteria, including mitotic count, sheeting, hypercellularity, small cells, prominent nucleoli, and spontaneous necrosis [16]. The 2021 Classification of CNS Tumors was revised to incorporate molecular criteria into the classification of meningiomas to form the basis of an integrated diagnostic framework. With these refined criteria, molecular alterations in the telomerase reverse transcriptase (*TERT*) promoter region and homozygous loss of the cyclin-dependent kinase inhibitor *(CDKN)2A/2B* are now classified with a Grade 3 designation [17,18,19,20]. *TERT* maintains telomere length and chromosomal stability and portends a worse prognosis when alterations are present in meningiomas [19]. *CDKN2A* encodes for the tumor suppressor p16(INK4A) and p14(ARF) proteins that inhibit cell growth and division [21]. Mutations in this gene are associated with inferior survival when observed in meningiomas [21,22]. Whereas Grade 1 meningiomas typically exhibit an indolent growth pattern, atypical CNS WHO Grade 2 meningiomas (4.3% of meningiomas) have a higher risk of recurrence [23]. Anaplastic CNS Grade 3 meningiomas account for 1.2% of meningiomas, are biologically aggressive and carry a risk of metastasis within and outside the CNS [2,23]. Grade 3 meningiomas of the papillary histologic subtype can harbor *PBRM1* loss [24]. Grade 3 meningiomas with either rhabdoid or papillary morphology can be associated with *BAP1* loss, which is often germline in nature [25].

Maximal safe resection is the cornerstone of treatment for tissue diagnosis and to provide symptomatic relief in meningiomas [12]. The extent of resection (EOR) is an integral component of correlative outcomes but may be prohibited by the tumor’s proximity to critical neurovascular structures. In 1957, the Simpson grade was developed to assess recurrence risk, though of late, its relevance in the modern era has been called into question [26,27]. For Grade 1 meningiomas, a gross total resection (GTR) can be curative and may obviate the need for additional treatment in the future. For Grade 2 meningiomas, adjunctive immediate or delayed radiation therapy (RT) may be indicated or safely omitted, but the optimal timing fueled by conflicting results remains and remains under debate [28,29]. To this end, the NRG BN-003 (NCT03180268) and ROAM/EORTC-1308 (ISRCTN71502099) Phase II investigations are currently underway to provide more insight into this unknown aspect [30]. For Grade 3 meningiomas, GTR and upfront RT (60 Gy delivered over 30 fractions) decrease local recurrence [31]. Dose escalation was investigated in the Phase II MARCIE trial for Grade 2 or 3 meningiomas with the addition of a carbon ion boost (18 Gy delivered over six fractions) to IMRT or fractionated stereotactic RT (50.4 Gy over 28 fractions). Although 3-year progression-free survival (PFS) and local control rates were >80%, the study was terminated due to increased toxicity [32].

Beyond surgery and radiation therapy, no effective therapies exist, and the prognosis is unvaryingly poor for refractory meningiomas with 6-month PFS (PFS-6) rates of 26–29% [33,34]. Numerous studies investigating different therapeutic approaches for recurrent meningiomas have been futile. Systemic therapies, including irinotecan, temozolomide, hydroxyurea, trabectedin, anti-hormonal therapy, and anti-angiogenic therapy, among others, have been extensively studied for use in recurrent meningiomas with marginal success [35,36,37,38,39,40,41,42,43,44,45]. One such anti-angiogenic therapy is bevacizumab, a biologic vascular endothelial growth factor (VEGF) inhibitor, which can be considered in the recurrent setting when surgery or radiation therapy is not feasible, with PFS-6 ranging from 43.8% to 87% [43,45,46]. However, anti-angiogenic therapy carries a risk of both non-fatal and fatal intratumoral hemorrhage, which can also be observed with sunitinib, a small-molecule biologic inhibitor [42,45]. 

Somatostatin receptor type 2 (SSTR), a G-protein-coupled receptor, is ubiquitously expressed in meningioma cells and regulates cell proliferation. For these reasons, SSTR as an actionable target has garnered considerable interest for the treatment of meningiomas (15, 16). SSTR is identifiable by immunohistochemistry or via positron emission tomography using [^68^Ga]-radiolabeled oxodotreotate (DOTATATE PET) [47,48]. SSTR-targeted therapy includes somatostatin analogs such as octreotide (an injectable somatostatin analog) and β-emitting [^177^Lu]-armed DOTATATE (Lutathera^®^) peptide receptor radionuclide therapy (PRRT) [49]. Octreotide activates SHP1 and SHP2 and inhibits the *PI3K*/*Akt* pathway, collectively mediating direct antitumor effects [50,51,52]. Octreotide monotherapy showed promise for use in meningiomas in early investigations with 44% PFS-6, but this success was not confirmed in subsequent studies [53,54,55,56,57]. This failure has been attributed to intracellular escape mechanisms [58]. To overcome these challenges, the addition of everolimus, a mammalian target of the rapamycin (*mTOR*) small-molecule inhibitor, to octreotide was studied in the CEVOREM trial [51]. *PI3K*/*Akt*/*mTOR* pathway targeting was justified by its putative role in the tumorigenesis of meningiomas that may stem from *NF2* inactivation [59,60,61,62,63,64,65]. Although the use of this combinatorial therapy suggested growth rate reductions, results from this trial were somewhat disappointing, with PFS-6 reaching only 55% [51]. Notably, none of the 14 enrolled patients with available analyzed tissue carried mutations in the *Pi3K* or *AKT* pathway [51].

A short follow-up of PFS-6 has been adopted as the current benchmark endpoint for refractory meningioma treatment trials [33,66,67]. However, the reliability of PFS-6 as a predictor of outcomes and biological behavior is debatable, given the frequent insidious growth rates observed in meningiomas [68]. Accordingly, PFS-6 as a point of reference shows modest outcomes, and the Response Assessment in Neuro-Oncology (RANO) Group recently advised that new benchmarks are warranted to inform of future trial success [34]. A three-dimensional volume growth rate (3DVGR) may represent a better predictor of outcome [52,69,70,71].

In this review, we aim to describe novel diagnostic tools and therapeutics for meningiomas. To identify relevant publications investigating innovative modalities and detect meningiomas and potential treatments for these tumors, searches of the literature were conducted in the PubMed database and on clinicaltrials.gov. The last search was completed on 26 February 2025. The search strategy adopted for this review using PubMed included the term “meningioma”. The search strategy adopted for this review using clinicaltrials.gov included the term “meningioma” with an “active” status. The references for accepted publications were analyzed for additional articles not identified in the initial search.

## 2. Novel Diagnostics

### 2.1. Grading Criteria

Meningioma grading and classification will continue to be enhanced with molecular features to offset the subjectivity of histopathologic interpretation. Additional guidance from cIMPACT-NOW released in the most recent version 8 addresses the subset of CNS WHO Grade 1 meningiomas that do not follow the natural course of a benign tumor and the subset of CNS WHO Grade 2 or 3 meningiomas that are not biologically aggressive [16,72]. The latest cIMPACT-NOW suggests that histomorphologic CNS WHO Grade 1 meningiomas harboring chromosomal aberrations with 1p deletion and 22q deletion and/or *NF2* oncogenic variants should be assigned as Grade 2 meningioma [16,73]. As we cultivate our understanding of these molecular underpinnings, the reliability of outcome predictions for meningiomas will also improve.

To date, there are no reliable predictors of response to radiation therapy. For this reason, improvements to meningioma risk stratification are warranted [74]. Chen et al. developed a 34-gene expression prognostic biomarker using unsupervised analysis to uncover patterns that alter risk in a discovery cohort of meningiomas [75]. Their results suggest that treatment decision-making, including response to radiation therapy, can be enhanced for ~30% of patients [75]. Their 34-gene expression biomarker improved risk stratification for Grade 2 meningiomas and could inform of those Grade 2 meningiomas that are suitable for close observation versus those that carry a risk of recurrence and may benefit from upfront radiation [75]. While this biomarker tool was validated on both retro- and prospective clinical samples, real-world decision-making based on this approach has yet to be investigated [75].

DNA methylation profiling attempts to overcome the limitations of the existing inter-rater variability in grading meningiomas. It is an objective approach to delineating meningiomas into discrete molecular groups with distinct biological behavior more reliably than the existing WHO grading criteria [76,77,78]. A DNA methylation-based classification model developed by Landry et al. was found to be superior to existing WHO 2021 grading criteria for predicting 5-year PFS in meningioma patients [79]. When integrated with EOR, DNA methylation profiling shows promise in predicting a subset of benign meningiomas that may recur [72]. Molecular modeling with DNA methylation, RNA expression, and copy number alterations may additionally advise on meningiomas that are likely to be radioresistant, including meningiomas within the proliferative molecular group or the *NF2* loss of function during hypoxia [78,80]. In stark contrast, meningiomas categorized into the *NF2* wildtype and immunogenic molecular groups appear to benefit from adjuvant radiation therapy [78]. This integrated model was recently validated and is available for public use [81]. However, a major disadvantage to DNA methylation profiling is its limited availability and protracted timing for results [76].

### 2.2. Imaging Modalities

[^68^Ga]DOTATATE PET has emerged as a formidable diagnostic tool for use in meningiomas. DOTATATE PET produces a high-imaging contrast due to high uptake in SSTR2-positive lesional tissue compared to the low background uptake in the brain and calvarium [82,83,84]. While it has yet to receive FDA approval for use in meningiomas, DOTATATE PET is approved and widely used for neuroendocrine tumors (NETs) in clinical practice [82,83,85]. Early investigations show that compared to MRI, DOTATATE PET has higher sensitivity and specificity for detecting meningiomas in both newly diagnosed and recurrent settings [86,87,88]. It excels at detecting viable tumor otherwise unrecognized or indistinguishable from dural scar on conventional MRI [89,90,91,92]. Moreover, cases of false positive detection of tumors with MRI can occur and lead to unnecessary treatment with radiation and unwarranted toxicity.

In addition to surveillance, DOTATATE PET may optimize the surgical planning approach in estimating the EOR, specifically in cases of intraosseous extension [93,94]. Moreover, it may offer benefits within radiation planning with more accurate target delineation volumes over conventional MRI that may overestimate lesional tissue in anatomically difficult locations or areas affected by post-operative scars [89,95,96,97]. Consequently, the added precision offered by DOTATATE PET can reduce the risk of unnecessary radiation exposure to normal tissue, including alopecia, optic neuropathy, and radiation necrosis, among others. Lastly, DOTATATE PET may provide predictive information for somatostatin-directed therapies and, when coupled with therapeutic radionuclides, can be harnessed for theranostics [58,98]. Based on these encouraging data, both the RANO Working Group and the National Comprehensive Cancer Network [99] guidelines have incorporated the consideration of DOTATATE PET for surveillance and surgical or radiation planning [100,101,102]. Advanced diagnostics for meningiomas are ongoing in active clinical trials (Table 1).

## 3. Novel Therapeutics

### 3.1. Small-Molecule Inhibitors

**Figure 1:**
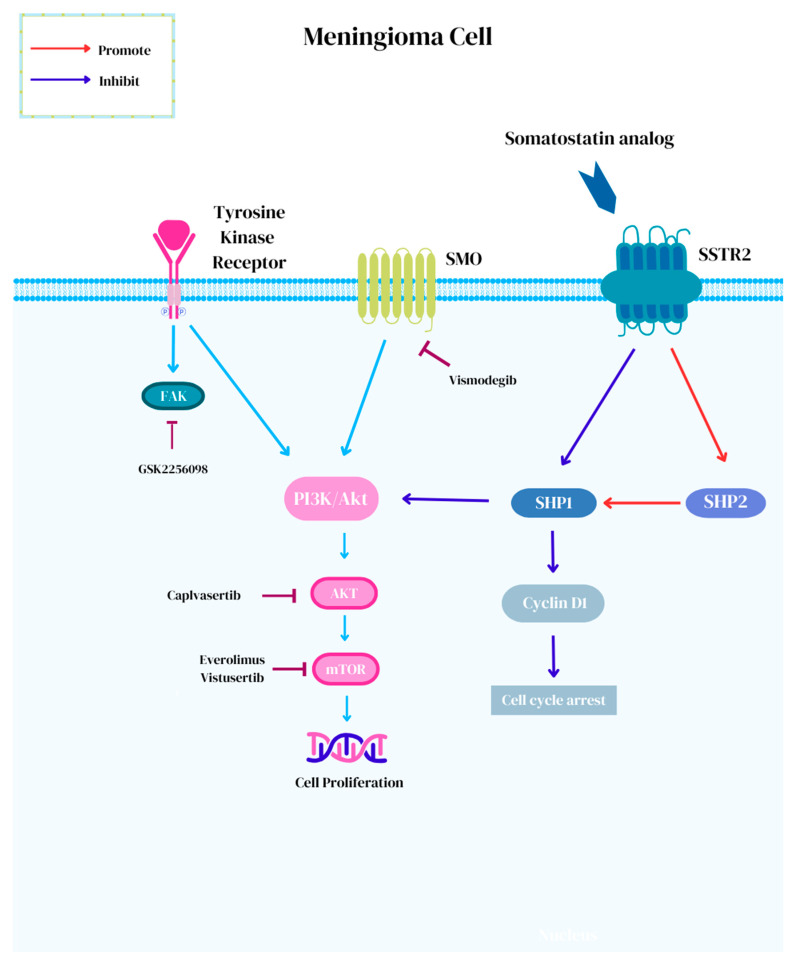
Novel small-molecule targeted therapies for the treatment of meningiomas.

Advances in molecular diagnostics have led to investigations for the subset of meningiomas with actionable targets. The European Society for Medical Oncology Scale for Clinical Actionability of Molecular Targets (ESCAT) assigns a score from I to V, which is used to describe the level of evidence for targeted therapy against meningioma [102]. ESCAT I is assigned to “ready for routine use” target–drug combinations with prospectively established clinical activity in meningioma. ESCAT II represents “investigational drugs” with known anti-meningioma activity but with an unknown magnitude of benefits, whereas ESCAT III and IV represent the efficacy of “hypothetical targets” in either other tumors (III) or in preclinical models (IV) [102]. To date, there are no molecular targets for meningiomas that have achieved a level of ESCAT I. At best, *mTOR* pathway activation and *NF2* alterations have attained an ESCAT II designation [102]. The Phase II Alliance A071401 (NCT02523014) trial is currently underway for recurrent/progressive meningiomas with driver mutations [103]. In this trial, meningioma patients harboring actionable targets are treated with small-molecule inhibitors, including neurofibromatosis-2 *(NF2*) mutations with GSK2256098, which is a focal adhesion kinase (FAK) inhibitor; *SMO* (ESCAT III) or *PTCH1* mutations with vismodegib; *CDK* (ESCAT IV) or *NF2* alterations with abemaciclib; and *AKT* (ESCAT III), *PI3K*, or *PTEN* mutations with capivasertib (Figure 1) [102,103,104].

*NF2* is a tumor suppressor on chromosome 22q12 encoding for the Merlin protein that plays an inhibitory role in the FAK, MAPK, and *PI3K*/*Akt*/*mTOR* signaling pathways that regulate cellular activity [59,64,65,102,105,106]. Approximately 50–60% of meningiomas harbor NF2 alterations resulting in a two-hit complete loss of function and are implicated in the tumorigenesis of sporadic meningiomas [107,108,109]. The presence of NF2 alterations can be present across meningioma of all grades (Grade 1= 37%, Grade 2 = 60%, and Grade 3 = 69%) and show genomic instability [110,111]. Early results for treatment with GSK2256098 in the NF2 arm of the Phase II Alliance A071401 (NCT02523014) trial showed partial response in 3% (1/36) and stable disease in 67% (24/36), with PFS-6 reaching 83% (10/12) in Grade 1 meningiomas and 33% (8/24) in Grade 2 and 3 meningiomas [103]. The most common toxicity associated with GSK2256098 is gastrointestinal adverse events [112].

Approximately 5% of meningiomas harbor *SMO* mutations [107,111]. *SMO* is a G-coupled protein receptor that encodes for a receptor that activates the sonic hedgehog signaling (SHH) pathway and results in subsequent differentiation and proliferation [102,107,111]. Meningiomas with *SMO* mutations generally follow a benign course [111]. Vismodegib, a small-molecule inhibitor of *SMO*, is efficacious for the treatment of basal cell carcinoma and CNS tumors, including SHH medulloblastomas [113,114,115,116,117]. Common adverse events include muscle spasms, alopecia, dysgeusia, diarrhea, fatigue, nausea, weight, and appetite loss [113,118]. In the NCI-MATCH ECOG-ACRIN Trial, meaningful responses were reported in meningioma patients with SMOPro641Ala and PTCHGlu947Ter alterations [119]. Resistance mechanisms to vismodegib most commonly seen among meningiomas, include neurotrophin signaling, downstream signaling changes, a lack of transduction of the SHH pathway, and the SMO^L412F^ mutation [111,118,120,121]. The suppressor of fused homolog (*SUFU*) is a negative regulator in the SHH pathway downstream to *SMO* [122]. As such, the upstream inhibition of *SMO* is therefore ineffective against SUFU mutant tumors [115,123].

Cyclin-dependent kinases (CDKs) regulate the cell cycle and apoptosis [22]. The homozygous deletion of CDKN2A/2B is now accepted as diagnostic criteria to classify meningiomas as WHO Grade 3, irrespective of histologic findings [17,22,68]. Abemaciclib is a brain-penetrating CDK4/6 inhibitor and has shown benefits in preclinical investigations [102,124,125]. The Phase II (NCT03071874) study investigating the use of vistusertib, the oral dual mTORC1/mTORC2 inhibitor in NF2-related schwannomatosis patients with either progressive or symptomatic meningiomas failed to meet the primary endpoint of a 20% volume decrease [126]. Six percent (1/8) achieved partial response and ninety percent (52/59) of patients achieved stable disease but this was poorly tolerated at 125 mg twice daily [126].

Phosphatidylinositol-4,5-bisphosphate 3-kinase catalytic subunit alpha (*PIK3CA*), *AKT* serine/threonine kinase 1 (*AKT1*), and phosphatase and TENsin homolog-deleted (*PTEN*) mutations were found to be capable of being targeted with capivasertib, an *AKT* inhibitor, in the NCI-MATCH trial. Meningiomas with *AKT1* mutations typically show chromosomal stability and are biologically benign [111]. The *AKT1* p.E17K mutation accounts for 10% of meningiomas and induces the constitutive activation of downstream oncogenic cellular effects by localizing from the cytoplasm to the plasma membrane [107,111,127]. Capivasertib has shown efficacy in hormone receptor-positive breast cancer and early investigations demonstrate response for the treatment of meningiomas [128,129,130]. Lastly, clear cell meningiomas harbor a loss of SMARCE1 [131]. SMARCE1 is associated with SWI/SNF chromatin remodeling and may be amenable to small-molecule inhibitors [131,132]. 

Driver mutations vary across meningiomas and may be lacking entirely. Accordingly, a universal targeted therapy such as SSTR2 would provide the advantage of treating virtually the entire cohort of progressive and high-risk meningiomas.

### 3.2. Somatostatin Analogs

Somatostatin analogs carrying high affinity to SSTR2, are an attractive treatment option for meningiomas and maintain a targeted yet generalizable and scalable approach to treatment [49]. Octreotide is an injectable somatostatin analog that activates SHP1 and SHP2 and inhibits the *PI3K*/*Akt* pathway, which collectively mediates direct antitumor effects [50,51,52]. Octreotide showed modest success for use in meningiomas in early investigations with 44% PFS-6, but this success was not reproducible and not substantiated in confirmatory studies [53,54]. This failure can potentially be attributed to intracellular escape mechanisms [58]. A nuanced approach with SSTR2-targeted theranostics has gained traction for use in meningiomas. [^177^Lu]DOTATATE (Lutathera ^®^) is a β-emitting radionuclide somatostatin analog that forms reactive oxygen species that trigger single-strand breaks within tumor DNA and ultimately, lethality [94,133]. Akin to antibody–drug conjugates, bystander, and crossfire effects may play a role in radionuclide therapy [134]. Following binding to the SSTR receptor and endocytosis, cytotoxicity affects adjacent tumor cells, but the restricted range can limit the crossfire effect and reduce the impact on neighboring normal brain tissue [49,134,135]. Additionally, the γ-photon-emitting capacity of [^177^Lu] can be leveraged for the pharmacokinetic imaging of the radiopharmaceutical in vivo [136]. [^177^Lu]DOTATATE achieved regulatory approval for use in advanced midgut NETs after showing prolonged PFS in the NETTER-1 Phase 3 trial [137]. Preliminary evidence demonstrates efficacy against meningiomas, which varies by grade with PFS-6 rates of 94%, 48%, and 0% in Grade 1, 2, and 3 SSTR2-positive meningiomas, respectively [49,135]. On this basis, PRRT is currently under investigational use in meningiomas and is jointly recommended within the EANM/EANO/RANO/SNMMI practice guidelines for consideration in cases of recurrence following standards of care given its low toxicity profile and proven efficacy in other cancers [58,135,138,139].

### 3.3. Brachytherapy

Brachytherapy is a form of implantable radiation therapy placed intraoperatively. Both iodine-125 (I-125) and cesium 131 (Cs-131) have been investigated for use in meningiomas and have shown a survival advantage [140,141,142]. However, the adoption of brachytherapy in clinical practice has been hindered by adverse events, including radiation necrosis, infection, wound dehiscence, and seed migration with high rates of reoperation [141,142,143]. Cs-131 carries the advantage of a reduced half-life compared to I-125 and the reduced risk of radiation necrosis [143]. GammaTile^®^ is a form of Cs-131 brachytherapy comprised of bioresorbable collagen tiles that conform to the surgical cavity to deliver uniform radiation without direct contact with normal brain parenchyma [144]. Results from a study investigating the use of GammaTile^®^ in meningioma patients showed that median overall survival was 26 months with 10.5% of patients experiencing radiation necrosis [145].

### 3.4. Systemic Radionuclide Therapy

With respect to ongoing trials, interim analysis from a Phase II study investigating [^177^Lu]DOTATATE in recurrent intracranial meningiomas showed encouraging findings, with 14% (2/14) of patients experiencing a > 25% reduction in tumor volume and 50% (7/14) achieving PFS-6 with stable disease as the best response (Table 2) [58]. The European Organization for Research and Treatment of Cancer (EORTC) is initiating the LUMEN-1 trial (NCT06326190), the first randomized study to compare [^177^Lu]DOTATATE versus the investigator’s choice (e.g., octreotide, everolimus, bevacizumab, sunitinib, hydroxyurea, or observation) in SSTR2-positive meningiomas. Braat et al. suggested that an intra-arterial route of administration for PRRT may saturate SSTR2 in meningiomas, resulting in increased tumor uptake and radiation dose absorption [49,146]. In Grade 1 or Grade 2 meningiomas, there is an apparent 6-month lag in antitumoral activity, but responses can persevere for up to 18 months following treatment initiation [147]. Myelosuppression is the most common treatment-related toxicity [135]. Other toxicities include fatigue, anemia, alopecia, and lymphopenia, which correlate with the number of prior systemic lines of therapy [47].

### 3.5. Immunotherapy

Immunotherapy has shown success and meaningful benefits in solid tumors, including melanoma and non-small-cell lung cancer, and is actively being studied in recurrent meningioma [148,149]. Though the programmed death-ligand 1 (PD-L1) is upregulated in meningiomas with higher expression levels rising with meningioma grade, the predictive role of PD-L1 expression remains notional [102,150,151]. The efficacy of immunotherapy may be limited by low-tumor mutational burden and the immunosuppressive tumor microenvironment [150,152]. Accordingly, immunotherapy has shown varied success in treating meningiomas [150,151]. In a Phase 2 study (NCT03279692), a PFS-6 rate of 48% and median PFS of 7.6 months was achieved in Grade 2 and 3 patients with recurrent meningioma treated with pembrolizumab [151]. Similarly, in another Phase 2 trial, prolonged survival was observed in 8% (2/25) of patients with recurrent Grade 2 and 3 meningioma with high mutational burden, though the primary endpoint was not met with a PFS-6 rate of 42.4%. [150].

## 4. Conclusions

To date, there are no effective therapies for refractory meningiomas. Recent advances in genetic alterations have gained traction in the grading of meningiomas and carry prognostic relevance. Newly uncovered molecular alterations, including TERT mutations and the homozygous loss of CDKN2A/2B, have improved upon the prior histopathologic grading schema. Growing evidence shows that chromosomal aberrations will further refine the grading schema for meningiomas. Additionally, the identification of driver mutations may reveal new therapeutic potential with small-molecule inhibitors. Further investigations with therapeutic targeted therapy, including [^177^Lu]DOTATATE and immunotherapy, may yield promise for future success in treating these tumors.

## 5. Future Directions

Artificial intelligence (AI) using radiomics may inform on recurrence risk [153]. Further development within the realm of radiomics, along with gene expression biomarkers, may also refine existing risk stratification models [75]. Currently, assertations regarding the use of AI in the diagnosis and management of meningiomas remain theoretical and speculative in nature but will certainly remain an area of active investigation in the future.

## Figures and Tables

**Table 1:** Current active diagnostic clinical trials for meningiomas in the United States.

Clinical Trial Identification	Primary Objective	Intervention	Intervention Category	Main Inclusion Criterion	Status (as of 2 January 2025)
					
NCT05139277 (Phase: NA)	Assess the CONVIVIO confocal endomicroscope’s ability to distinguish between normal and abnormal tissue intraoperatively	CONVIVO system	Diagnostic test	Intracranial meningiomas and other brain tumors	Recruiting
NCT06014905 (Phase 1)	Evaluate the feasibility of using hyperpolarized [^13^C] MR imaging to non-invasively characterize aggressive tumor behavior in patients with meningioma	Hyperpolarized carbon [^13^C] pyruvate + MRI	Diagnostic Test	Intracranial WHO Grade 1–3 meningioma with at least 1 cm of gadolinium enhancement	Recruiting
NCT06650163 (Phase 1)	Assess zirconium [^89^Zr] crefmirlimab berdoxam and immuno-PET’s ability to identify areas of immune cell activity in brain tumors	[^89^Zr]Crefmirlimab berdoxam	Diagnostic Test	Meningiomas of any grade and other brain tumors	Recruiting
NCT04298541 (Phase 2)	Compare [^68^Ga]DOTATATE PET/CT or PET/MR to [^68^Ga]DOTATOC PET/CT in meningioma patients	[^68^Ga]DOTATATE, [^68^Ga]DOTATOC	Drug	WHO Grade 1–3 meningioma	Not yet recruiting
NCT06439420 (Phase 2)	Evaluate the efficacy of Cognitive Behavioral Therapy for Insomnia (CBT-I) in patients with primary brain tumors	CBT-I	Behavioral	Meningiomas and other primary brain tumors of all WHO grades	Recruiting
NCT04743310 (Phase 2)	Study the use of tozuleristide and Canvas imaging systems during brain tumor resections	Tozuleristide and Canvas imaging system	Drug and Device	High-grade meningioma and other primary brain tumors	Recruiting
NCT06377371 (Phase 4)	Evaluate the feasibility of using [^64^CU]DOTATATE for intraoperative tumor detection	Brain imaging with [^64^Cu]DOTATATE	Diagnostic Test	WHO Grade 1–3 meningioma	Recruiting
NCT04081701 (Phase 4)	Evaluate the use of [^68^Ga]DOTATATE PET/MRI in diagnosing and managing patients with somatostatin receptor-positive (SSTR-positive) CNS tumors	[^68^Ga]DOTATATE-PET/MRI	Diagnostic Test	WHO Grade 1–3 meningioma and other SSTR-positive brain tumors	Recruiting

**Table 2:** Current active therapeutic clinical trials for meningiomas in the United States.

Clinical Trial Identification	Primary Objective	Intervention/ Treatment	Intervention/ Treatment Category	Main Inclusion Criterion	Status (as of 2 January 2025)
					
NCT06557512 (Phase: NA)	Assess the safety and efficacy of hypofractionated stereotactic radiosurgery after GTR of intermediate-risk meningioma	Hypofractionated stereotactic radiosurgery	Radiation	WHO Grade 2 or recurrent WHO Grade 1 meningioma	Recruiting
NCT04541082 (Phase 1)	Assess the safety and tolerability of oral ONC206 in patients with recurrent primary brain tumors	ONC206	Drug	Recurrent meningiomas and other primary brain tumors	Recruiting
NCT03604978 (Phase 1/2)	Evaluate the side effects and best dose of nivolumab combined with multi-fraction stereotactic radiosurgery with or without ipilimumab	Nivolumab + multi-fraction stereotactic radiosurgery ±ipilimumab	Drug and Radiation	Recurrent WHO Grade 2–3 meningioma	Recruiting
NCT02693990 (Phase 1/2)	Investigate the feasibility of increased-dose intensity-modulated proton therapy (IMPT) for treatment of meningioma	IMPT	Radiation	WHO Grade 2–3 meningioma	Recruiting
NCT05278208 (Phase 1/2)	Investigate the safety and efficacy of Lutathera in patients with progressive or recurrent high-grade brain tumors and meningiomas with uptake on DOTATATE PET	Lutathera^®^ ([^177^Lu]DOTATATE)	Drug	Progressive, recurrent, or refractory meningioma of any WHO grade and WHO Grade 3–4 primary CNS tumors	Recruiting
NCT05425004 (Phase 2)	Assess the efficacy of cabozantinib for patients with recurrent or progressive meningioma	Cabozantinib	Drug	Recurrent or progressive WHO Grade 1–3 meningioma	Recruiting
NCT05940493 (Phase 2)	Investigate how abemaciclib works in treating patients with Grade 3 meningioma	Abemaciclib	Drug	Intracranial WHO Grade 3 meningioma or lower-grade meningioma that has progressed to WHO Grade 3	Not yet recruiting
NCT02847559 (Phase 2)	Evaluate the effects of bevacizumab combined with Optune-delivered electric field therapy on meningiomas	Bevacizumab + electric field therapy (using Optune device)	Drug, Device	WHO Grade 2 or 3 meningioma	Recruiting
NCT04082520 (Phase 2)	Assess the efficacy of Lutathera in treating patients with inoperable and progressive meningioma after external beam radiation therapy	[^177^Lu] Dotatate	Drug	Inoperable and progressive WHO Grade 1–3 meningioma	Recruiting
NCT04659811 (Phase 2)	Assess the efficacy of stereotactic radiosurgery and pembrolizumab for treating patients with recurrent meningioma	Pembrolizumab, stereotactic radiosurgery	Drug, Radiation	Recurrent or progressive WHO Grade 2–3 meningioma	Recruiting
NCT02523014 (Phase 2)	Study how vismodegib, FAK inhibitor GSK225609, capivasertib, and abemaciclib work in treating progressive meningioma	Vismodegib, FAK inhibitor GSK2256098, capivasertib, abemaciclib	Drug	Progressing WHO Grade 1–3 meningioma	Recruiting
NCT05023018 (Phase 2)	Assess the safety, pharmacokinetics, and efficacy of NEO100 for treating residual high-grade meningioma	NEO100 (perillyl alcohol)	Drug	Residual, progressive, or recurrent WHO Grade 2–3 meningioma	Recruiting
NCT04278118 (Phase 2)	Study the efficacy of hypofractionated protons or photon radiation therapy in treating benign brain tumors	Hypofractionated radiation therapy, photon beam radiation therapy, and proton beam radiation Therapy	Radiation	Meningiomas of all grades and other benign primary brain tumors	Recruiting
NCT06132685 (Phase 2)	Evaluate the efficacy of tapering doses of dexamethasone after a craniotomy in patients with brain tumors	Dexamethasone	Drug	WHO Grade 1–3 meningiomas and other brain tumors	Recruiting
NCT05130866 (Phase 2/3)	Study the safety and effectiveness of REC-2282 in treating progressive NF2-mutated meningiomas	REC-2282	Drug	Progressive WHO Grade 1–3 meningioma in patients with either NF2 mutation, NF2 disease, or at least one other NF2-related tumor	Recruiting
NCT03180268 (Phase 3)	Evaluate the efficacy of radiation therapy after GTR of Grade 2 meningioma	Radiation therapy	Radiation	WHO Grade 2 meningioma	Recruiting

GTR: gross total resection; PET: positron emission tomography; FAK: focal adhesion kinase; NF2: neurofibromatosis type 2.

## Abbreviations

The following abbreviations are used in this manuscript:

Abbreviation	Definition
AI	Artificial intelligence
*AKT1*	*AKT* serine/threonine kinase 1
*BAP1*	*BRCA1* associated protein 1
CDK	Cyclin-dependent kinase
CDKN	Cyclin-dependent kinase inhibitor
CNS	Central nervous system
CT	Computed tomography
EOR	Extent of resection
EORTC	European Organization for Research and Treatment of Cancer
ESCAT	European Society for Medical Oncology Scale for Clinical Actionability of Molecular Targets
*FAK*	Focal adhesion kinase
GEP	Gastroenteropancreatic
GTR	Gross total resection
IMRT	Intensity-modulated radiation therapy
MRI	Magnetic resonance imaging
NF2	Neurofibromatosis type 2
NCT	National Clinical Trial
NCCN	National Comprehensive Cancer Network
NET	Neuroendocrine tumor
OS	Overall survival
*PBRM1*	Polybromo-1
PD-L1	Programmed death-ligand 1
DOTATATE PET	Positron emission tomography with [^68^Ga]DOTATATE
PFS	Progression-free survival
*PI3K*	Phosphoinositide 3-kinase
PRRT	Peptide receptor radionuclide therapy
*PTEN*	Phosphatase and TENsin
RANO	Response Assessment in Neuro-Oncology
RECIST	Response Evaluation Criteria in Solid Tumors
RT	Radiation therapy
SHH	Sonic hedgehog signaling
SMARCE1	SWI/SNF-related matrix-associated actin-dependent regulator of chromatin subfamily B member 1
*SMO*	Smoothened
SUFU	Suppressor of fused homolog
3DVGR	Three-dimensional volume growth rate
*TERT*	Telomerase reverse transcriptase
WHO	World Health Organization
